# Preparation of culture plates demonstrating anti-Staphylococcus aureus activity of Penicillium sp. and their use as teaching materials

**DOI:** 10.1099/acmi.0.001129.v3

**Published:** 2026-06-12

**Authors:** Tomoe Ichikawa, Yoshio Ishibashi

**Affiliations:** 1 Department of Microbiology and Immunology, Faculty of Pharmaceutical Sciences, Shonan University of Medical Sciences, Yokohama, Japan

**Keywords:** Fleming, microbiology, penicillin, *Penicillium*

## Abstract

Penicillin is recognized as one of the world’s first antibiotics, originally derived from *Penicillium* sp. However, few students have directly observed the antibacterial activity of *Penicillium* sp. against bacterial pathogens. Therefore, we hypothesized that observing this phenomenon could enhance the interest of pharmacy students in micro-organisms and antibiotics. First, we selected a *Penicillium* strain exhibiting strong antibacterial activity and established a reproducible method to prepare culture plates showing clear inhibition of *Staphylococcus aureus* growth. During a microbiology practical course, students observed these prepared plates, and a questionnaire survey of university students was conducted before and after the session. Students were asked whether observing this antibacterial activity increased their interest in antibiotics and aided their retention of knowledge about antibiotics. Over 80% of respondents answered favourably, suggesting that incorporating images of plates showing *Penicillium*-mediated antibacterial activity into teaching would improve students’ interest in microbiology and antibiotics.

## Data Summary

The authors confirm that the data supporting the findings of this study are available within the article and its supplementary materials.

## Introduction

Penicillin is one of the world’s first antibiotics, and its discovery by Alexander Fleming is widely regarded as serendipity. Fleming discovered that *Staphylococcus* colonies near a mould contaminant were dying [1]. From this observation, he deduced that the mould, identified as *Penicillium* sp., was producing antibacterial substances. This conditioned medium of fungus was known as ‘penicillin’, and detailed experiments were subsequently conducted using the conditioned media and cultured cells to confirm this activity [2,3]. The above anecdote is often introduced in microbiology classes at universities. Although Fleming’s original paper includes a photograph of an agar plate showing the antibacterial effect of filamentous fungi, it is in black and white. Today, there are few photographic resources available in books or online, and most available images lack specific information about the micro-organisms involved.

Experiments demonstrating antibiotic effects are sometimes conducted as part of practical training at universities. However, when antibacterial activity is observed in practical training, it typically involves purified drugs, such as certain penicillin antibiotics, in MIC assays or disc diffusion tests. In the microbiology practical training at our university, students perform drug susceptibility testing using standard antibiotics, but they rarely have the opportunity to observe agar plates on which fungi themselves produce antibacterial compounds. It is believed that visual observation of the production of antibacterial substances by *Penicillium* spp. could spark student interest in antibacterial drugs and enhance understanding of their functions. However, the long cultivation time required for fungal culture, especially filamentous fungi, often prevents their incorporation into practical training. Therefore, we developed culture plates that are similar to those reported by Fleming to visually demonstrate the antibacterial activity of *Penicillium* sp. for use in teaching. We first selected a *Penicillium* strain that produces antibacterial substances. Subsequently, we optimized a culture method suitable for efficient observation, even in laboratories not accustomed to handling filamentous fungi. In addition, a questionnaire survey was administered before and after practical training using photographs of these plates to evaluate the educational impact on student learning and engagement.

## Methods

### Experiments using micro-organisms to prepare specimens for observation

The *Penicillium* strains and *Staphylococcus aureus* used in this study are classified as biosafety level 1 and 2 organisms, respectively. All experiments involving these micro-organisms were performed in a biosafety cabinet. Equipment and culture media were sterilized using an autoclave (high pressure steam sterilizer) before disposal. Individuals performing the experiments, including teachers and students, were required to be proficient in microbiological techniques and comply strictly to general laboratory work guidelines. The plates with microbial growth were sealed with parafilm to prevent lid displacement and then provided to students for practical training.

### Strains and media

Penicillin was first found by Sir Alexander Fleming, and the fungus producing it was identified as *Penicillium notatum*, later reclassified as *Penicillium chrysogenum*, and is now recognized as *Penicillium rubens* [4]. The names of the *Penicillium* strains used in this study are not standardized across different microbial distribution centres. Therefore, we refer to the strain names as listed by the American Type Culture Collection (ATCC).

*P. rubens* ATCC 9783 (NRRL 792=CBS 129667=NBRC 32029), *P. rubens* ATCC 10002 (NRRL 66087=CBS 277.47=NBRC 4626), *P. chrysogenum* ATCC 10106 (NRRL 807=CBS 306.48=NBRC 32030), *S. aureus* ATCC 29213 (NBRC 15035), *S. aureus* ATCC 25923 (NBRC 14462), *S. aureus* ATCC 6538 (NBRC 13276) and *S. aureus* type strain ATCC 12600 (NBRC 100910) were used in this study. The *Penicillium* strains selected have been reported to produce penicillin. All strains were purchased from the Biological Resource Center, National Institute of Technology and Evaluation (NBRC, Tokyo, Japan) [4,5]. The *S. aureus* strain was cultured on nutrient agar (NA) medium (Eiken Chemical Co., LTD., Tokyo, Japan) at 37 °C. *Penicillium* strains were precultured on potato dextrose agar (PDA) (Merck KGaA, Darmstadt, Germany) or Yeast Mold (YM) agar (Becton, Dickinson and Company, NJ, USA) plates at 22 °C.

### Growth inhibition tests using *Penicillium* strains

*Penicillium* strains were precultured on PDA or YM agar plates for 6–9 days, and the *S. aureus* ATCC 29213 strain was cultured on NA for 24 h. Following incubation, *S. aureus* cells were suspended in 0.85% physiological saline solution (PSS) and adjusted to an optical density of 0.05 at 600 nm (OD600). This suspension was further diluted 500-fold using PSS, and then, 100 µl of the diluted suspension was spread evenly onto 9 cm NA plates using a sterile bacterial spreader. Alternatively, an appropriately diluted *S. aureus* suspension was streaked onto the medium using a sterile cotton swab. Some students failed to recognize the presence of bacteria when bacteria grow confluently across the entire plate. When culturing the bacteria to a confluent state, leaving a portion of the medium free of bacteria helps students understand the areas where bacteria have grown. Thus, in this study, a sterile cotton swab was used to smear the bacteria, leaving a surrounding area free of bacteria. Once the surface of the medium had dried, the *Penicillium* strains were inoculated at the centre of the plate.

*Penicillium* strains were inoculated using two methods: in the first, only the fungi were transferred using an L-shaped platinum colony hook; in the second, the *Penicillium* strains were transferred along with the precultured medium using a sterile microspatula. The use of a platinum hook to inoculate the mycelium from the underside against the agar medium reduces contamination [6]. However, in this study, *Penicillium* sp. was inoculated from the top side of the agar medium even when using platinum hooks because the number of fungal cells that could be inoculated using this method was limited (Fig. S1, available in the online Supplementary Material). The microspatula is not an instrument for fungal inoculation, but it is used as an auxiliary tool to excise filamentous mycelia and to inoculate and preserve filamentous fungi [7,8]. In this study, a sterilized microspatula was used to press and cut the filamentous mycelia along with the medium. The inoculation method is captured and explained with photos in Fig. S1. All experimental procedures were conducted in a Class IIA biosafety cabinet. The plates were incubated at different temperatures, such as 20 °C, 22 °C and 25 °C. After the start of culture in air, the inhibition zone was observed, and its size was measured on the third, fourth and fifth days. The diameter of the inhibition zone was measured using a line rule. For each condition, three replicate plates were prepared, and the average inhibition diameter and sd were calculated.

In comparing susceptibility to antibacterial compounds among multiple *S. aureus* strains, the bacterial loads described by the EUCAST disc diffusion method were used [9]. In particular, *S. aureus* culture prepared to McFarland 0.5 was streaked onto a medium using a sterile cotton swab, and then, *P. rubens* was inoculated and cultured at 20 °C for 5 days or 35 °C for 1 day. In addition to ATCC 29213 (a weak beta-lactamase-producing strain), three additional *S. aureus* strains were also used: ATCC 25923 (a beta-lactamase-negative strain), ATCC 6538 and ATCC 12600 [10]. ATCC 12600 is sensitive to penicillin [11].

Welch’s ANOVA followed by Dunnett’s T3 multiple comparisons test was used to compare groups. Statistical analyses were performed using GraphPad Prism (GraphPad Software, Inc., USA).

### Student surveys

#### Participants and data collection

The subjects were students in the 6-year Faculty of Pharmacy. At our university, the microbiology class is offered to second-year students, while the practical training session on micro-organisms is offered to third-year students. Interview-1 was conducted with second-year students before their practical training session on micro-organisms, and Interview-2 was conducted with third-year students after they completed their practical course.

### Interview-1

An anonymous seven-question survey was administered via Google Forms to 81 second-year students who had completed a lecture on antibiotics. A simplified version of the questionnaire is presented in Table 1, and the full version is provided in Appendix S1. Interview-1 was conducted in November 2024.

**Table 1.** Questionnaire items and answers (*n*=33)

Item no.	Questionnaire items. For the full text, see Appendix S1.	Answer choices		No. of selected choices (%)	Total no.
1	Do you know the story of the discovery of penicillin by Fleming?	Yes		15 (45.5)	33
		No		18 (54.5)	
2	Have you ever seen *Penicillium* sp. inhibiting the growth of bacteria?	Never		16 (48.5)	33
		Only seen in videos or photos		16 (48.5)	
		Only seen it on an actual agar plate		1 (3.0)	
		Seen it both on an actual agar plate and in videos or photos		0 (0)	
3	Please choose the image you think best demonstrates the antibacterial activity of *Penicillium* sp.	A	to view the image see Figure 1	13 (40.6)	32
		B		3 (9.4)	
		C		16 (50.0)	
4	Please choose the image you think illustrates the second best antibacterial effect	A	to view the image see Figure 1	10 (30.3)	33
		B		9 (27.3)	
		C		14 (42.4)	
5	Do you want to look at the actual agar plates used in the photographs shown in Q3 and Q4?	Yes		16 (48.5)	33
		Rather yes		12 (36.4)	
		Neither yes nor no		4 (12.1)	
		Rather no		1 (3.0)	
		No		0 (0)	
6	Do you think looking at the experimental results of antibacterial activity assays involving *Penicillium* sp. would increase interest in antibiotics or help students retain knowledge?	Yes		18 (54.5)	33
		Rather yes		11 (33.3)	
		Neither yes nor no		4 (12.1)	
		Rather no		0 (0)	
		No		0 (0)	
7*	Do you think the effectiveness of ‘looking at the antibacterial activity of *Penicillium* sp.’ is different between observing the actual agar plate in which the bacteria and fungi are cultured and looking at a photograph of it?	Photos are more effective		2 (6.9)	29
		Effectiveness is the same whether the actual agar plate is shown or a photo of it		7 (24.1)	
		Actual agar plate is more effective		20 (69.0)	

*Only students who selected ‘Yes’ or ‘Rather yes’ in Q6 answered this question.

**Fig. 1.**
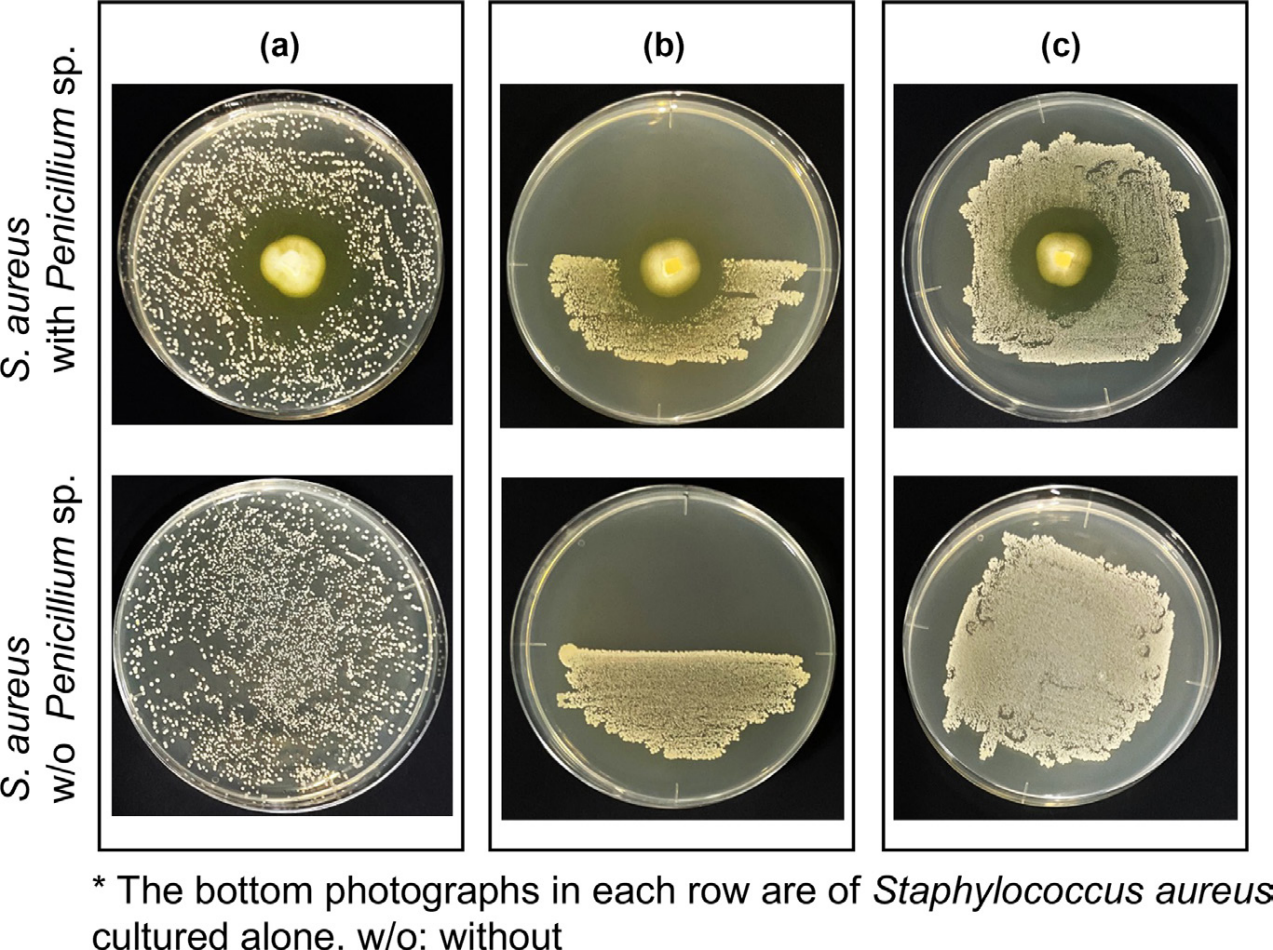
Preparation of culture plates for the photographs used in student interviews. *S. aureus* was suspended in PSS and adjusted to OD600 of 0.05. For plate A, this suspension was diluted 500-fold, and 100 µl was spread evenly across the plate. For plates B and C, the suspension was diluted fivefold and streaked using a sterile cotton swab. After the medium surface of the medium dried, *P. rubens* ATCC 10002 was inoculated at the centre of the plate using a microspatula. All plates were incubated at 20 °C for 5 days. The top panels show cocultures of *P. rubens* and *S. aureus*, while the bottom photographs in each row show *S. aureus* cultured alone. w/o: without. This figure was used in student interviews.

In Interview-1, students were asked to select the photograph they believed showed the strongest antibacterial activity, based on three sets of images showing similar inhibition zone sizes (Fig. 1). These images, derived from culture plates prepared in this study, were presented to students without any accompanying information about the culture conditions. Photos A, B and C from Table 1 are displayed in Fig. 1 (Appendix S1 for the full text and photos). Each plate was cultured with *P. rubens* ATCC 10002 at 20 °C for 5 days under the following culture conditions: A: *S. aureus* was smeared using a sterile bacterial spreader, and *P. rubens* was inoculated with a spatula. B: *S. aureus* was smeared in a semicircular shape using a cotton swab, and *P. rubens* was inoculated with a spatula. C: *S. aureus* was smeared in a circular shape using a cotton swab, and *P. rubens* was inoculated with a spatula.

### Interview-2

Anonymous seven-question interviews were conducted using Google Forms with 69 third-year students following completion of their microbiology practical course. An abbreviated version of the interview guide is provided in Table 2, and the full questionnaire is available in Appendix S1. Interview-2 was conducted in June 2025. This survey also included a question asking participants to select the photo they believed demonstrated the strongest antibacterial effect, using the same three sets of images (Fig. 1) as in Interview-1.

**Table 2.** Questionnaire items and answers (*n*=20)

Item no.	Questionnaire items. For the full text, see Appendix S1.	Answer choices		No. of selected choices (%)	Total no.
1	Do you remember looking at the photos depicting the antibacterial activity of *Penicillium* sp. in the second-year survey?	Yes		12 (60.0)	20
		No		5 (25.0)	
		I didn't answer the questionnaire when I was a second-year student		3 (15.0)	
2	During your practical training, did you see the actual Petri dishes?	Yes*		18 (90.0)	20
		No**		2 (10.0)	
3	Please choose the image you think best demonstrates the antibacterial activity of *Penicillium* sp.	A	to view the image see Figure 1	8 (44.4)	18
		B		1 (5.6)	
		C		9 (50.0)	
4	Please choose the image you think illustrates the second best antibacterial effect	A	to view the image see Figure 1	5 (27.8)	18
		B		7 (38.9)	
		C		6 (33.3)	
5	Do you think looking at the experimental results of antibacterial activity assays involving *Penicillium* sp. would increase interest in antibiotics or help students retain knowledge?	Yes		9 (50.0)	18
		Rather yes		6 (33.3)	
		Neither yes nor no		3 (16.7)	
		Rather no		0 (0)	
		No		0 (0)	
6	Do you think the effectiveness of ‘looking at the antibacterial activity of *Penicillium* sp.’ is different between observing the actual agar plate in which the bacteria and fungi are cultured and looking at a photograph of it?	Photos are more effective		6 (42.9)	14
		Effectiveness is the same whether the actual agar plate is shown or a photo of it		1 (7.1)	
		Actual agar plate is more effective		7 (50.0)	
7	Could you please tell me why you didn't look at the actual agar plates?				0

*If you choose this answer, go to Q3.

**If you choose this answer, go to Q7.

### Ethics approval

This study was approved by the ethics committee of our university (Approval number: 24–012) and conducted using an opt-out system. Only those who wanted to participate completed the questionnaire. The students were informed, verbally and through written notices on our university’s internal website, about the voluntary nature of participation that refusal to participate would not result in any disadvantages and they had the opportunity to opt out at any time after participating. In this study, only those who consented could respond to the questionnaire anonymously; thereby, responding to the questionnaire was considered as providing written consent. Research consent denial and withdrawal forms were also distributed. Students who did not submit the consent denial form could still express their disagreement by not completing the survey.

### Statistical analysis for surveys

Fisher’s exact test was used for categorical data and was performed using the JMP Pro 18 software. Statistical significance was defined as *P*<0.05.

## Results

### Experiments using micro-organisms to prepare specimens for observation

**Fig. 2.**
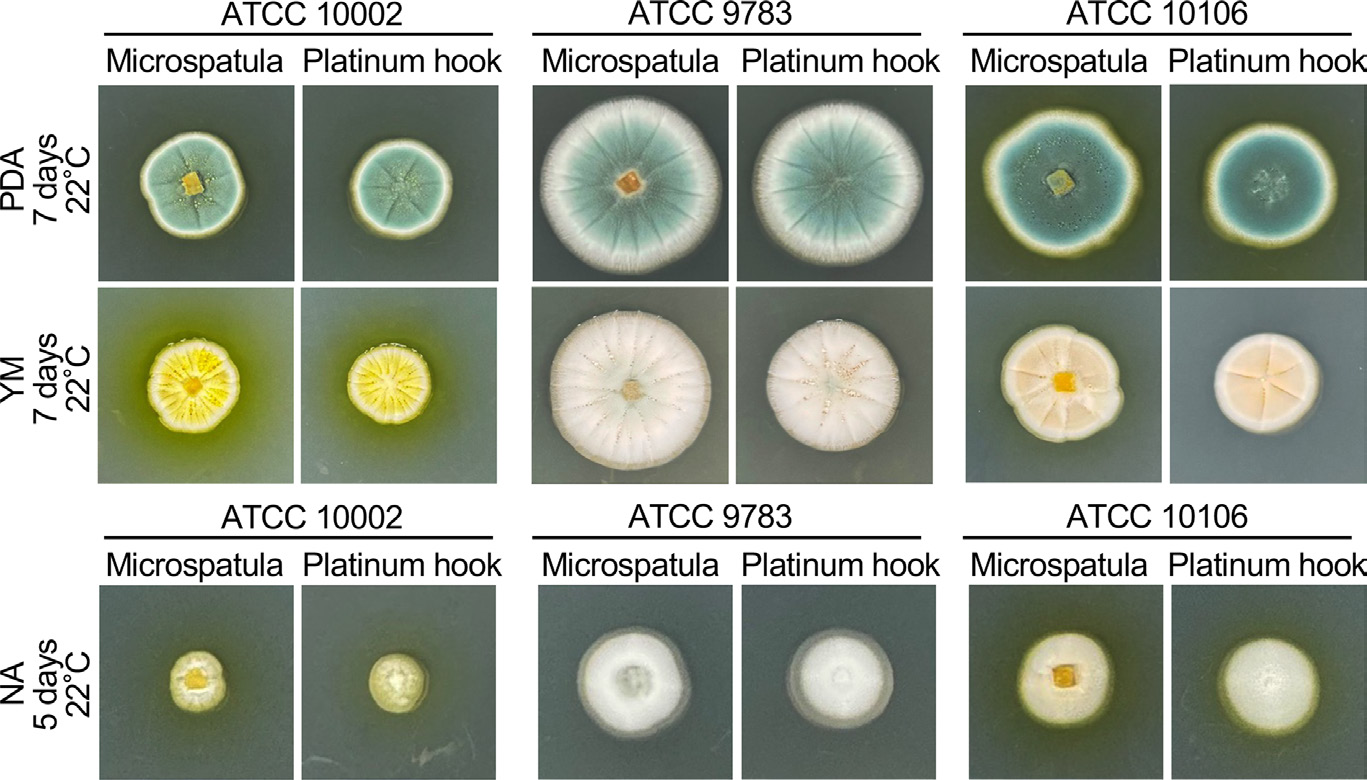
Colony colouration and growth characteristics of the three *Penicillium* strains on different media. *Penicillium* colonies grown on PDA medium were blue-green (top photo). *Penicillium* colonies grown on YM agar medium were milky white, yellow or orange (middle photo). Colonies grown on NA medium, typically used for *S. aureus* (bottom photo), showed comparable diameters when inoculated with a microspatula or platinum hook.

**Fig. 3.**
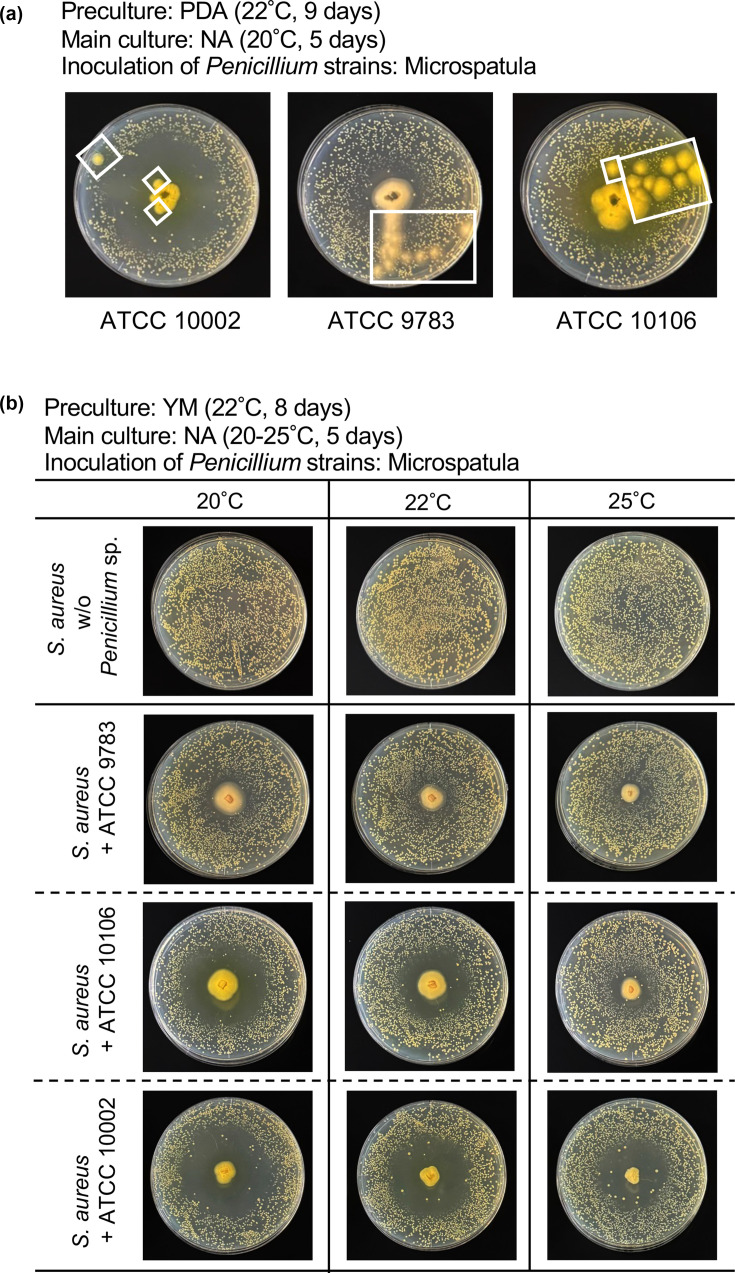
Inhibition zones formed by three *Penicillium* strains against *S. aureus*. Photos were obtained from the back of the plates. (a) Experimental results using PDA as the preculture medium. Penicillium strains were inoculated using a microspatula and cultured. White square indicates *Penicillium* proliferation at unintended locations. (b) Differences in antibacterial activity among the three *Penicillium* strains and differences in inhibition zone sizes depending on incubation temperature. YM agar was used for preculture, and *Penicillium* strains were inoculated using a microspatula. The photo was obtained from behind the plate. The incubation temperatures were compared at 20 °C, 22 °C and 25 °C. The top panel shows a control plate with only *S. aureus*, without *Penicillium* inoculation. w/o: without.

*Penicillium* sp. is widely known as a blue or green mould; however, its colouration varied depending on the growth medium (Fig. 2). PDA is commonly used for culturing filamentous fungi, while YM agar is considered suitable for yeast and filamentous fungi [12,13]. *Penicillium* strains grown on PDA medium turned blue-green, whereas *those* grown on YM agar medium did not. As conidia are generally green [14], this result suggests that sporulation was reduced on YM medium. In our experiments, when the *Penicillium* strains were precultured on PDA and then inoculated using a microspatula, conidia were unintentionally scattered across the plate (Fig. 3a). In contrast, preculture on YM medium resulted in no conidium dispersion upon inoculation, indicating higher conidium formation when cultured on PDA medium (Fig. 3b). As mentioned above, although PDA is a commonly used medium for culturing filamentous fungi, it may not be ideal for producing clean, visually appealing specimens owing to the scattering of conidia. Importantly, when YM agar medium was used for precultivation of *Penicillium* strains, the inhibition zone was equal or even larger than that of the precultured *Penicillium* strains on PDA (Fig. 3a, data not shown), indicating that YM agar medium was more suitable for this experiment.

**Fig. 4.**
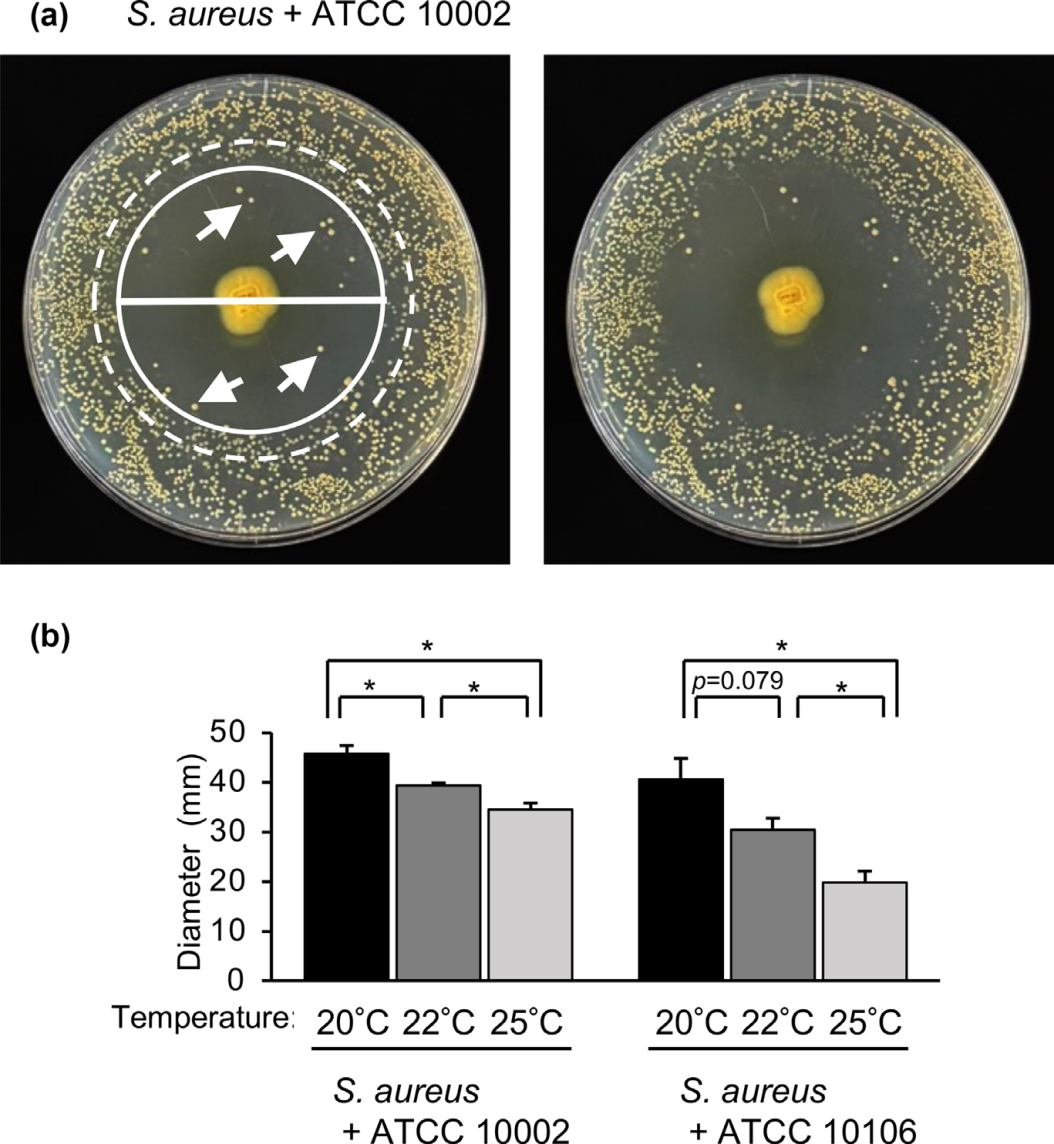
Inhibition zone measurement. (a) Inhibition zones and *S. aureus* colonies exhibiting induced resistance. The left and right panels show the same photo. White solid circle: growth inhibition zone. Region between the solid and dotted white circles: *S. aureus* colonies undergoing lysis. White solid line: diameter of the growth inhibition zone. Arrow: example of colonies exhibiting acquired induced resistance. w/o: without. (b) Graph of inhibition zone diameters. The diameter of the inhibition zone was measured at each incubation temperature of 20 °C, 22 °C and 25 °C (*n*=3). *: *P*<0.05.

Of the three strains tested, *P. rubens* ATCC 10002 exhibited the strongest antibacterial activity, followed by ATCC 10106. With respect to temperature, incubation at 20 °C produced the strongest activity with a large inhibition zone (Figs3b  4b). Considering that ATCC 29213 is a weak *β*-lactamase producer, the colonies found within the inhibition zone may represent induced resistance because of the selective pressure from the compounds or may represent heteroresistance, with the degree of resistance varying among individual *S. aureus* cells (Fig. 4a, white arrow). Two inoculation methods were compared: one using a microspatula and the other using a platinum hook. The inhibition zone was clearly larger when the microspatula was used, likely because it allowed for the transfer of a greater number of *Penicillium* cells.

**Fig. 5.**
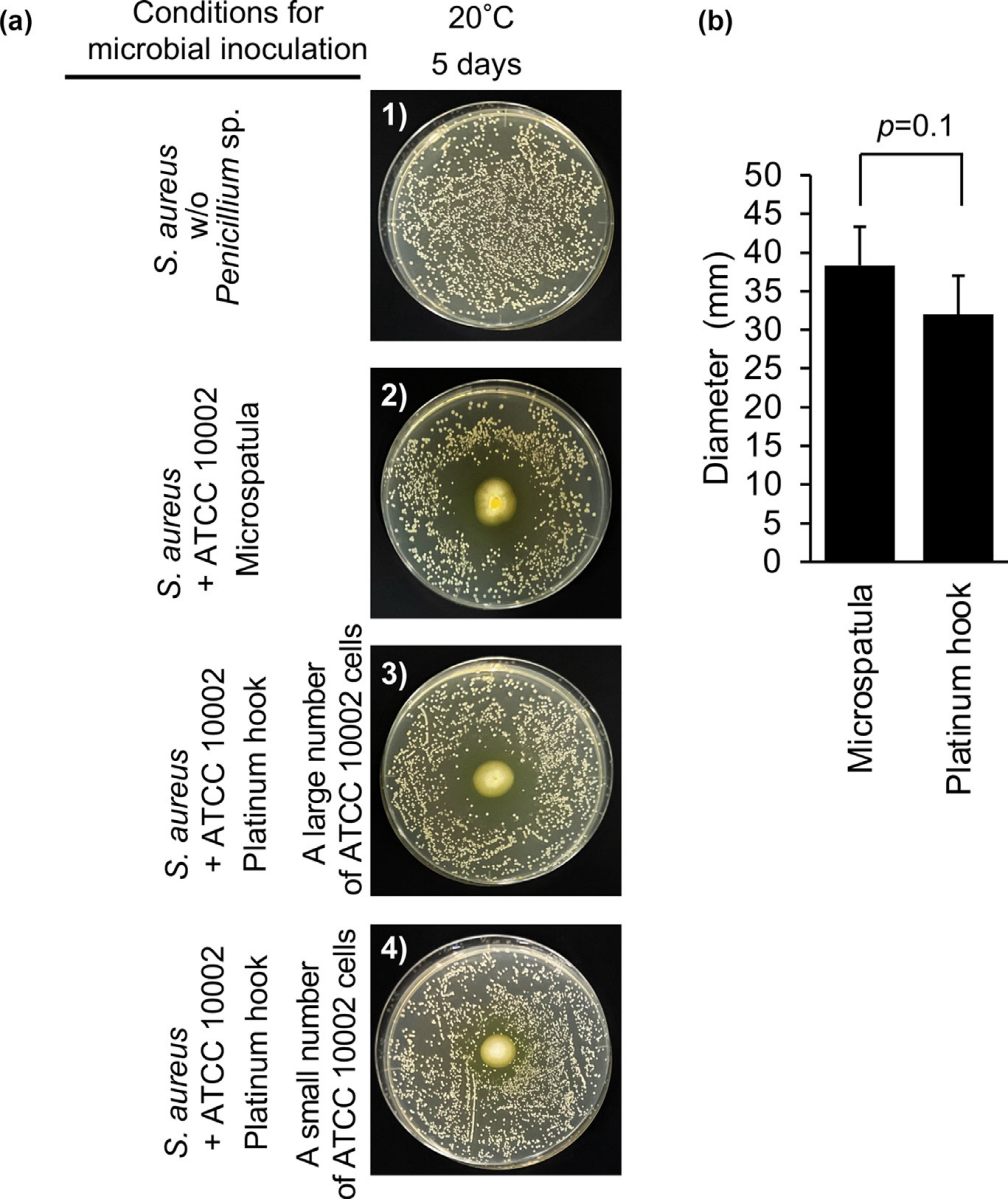
Inoculation method and inhibition zone. (a) Inhibition zone of *P. rubens* ATCC 10002 against *S. aureus* inoculated using a microspatula or platinum hook. Conditions for microbial inoculation are shown to the left of each plate photo. Photographs were obtained from the plate surface. (1) Control plate with only *S. aureus*. (2) Plate inoculated with *P. rubens* ATCC 10002 using a microspatula. (3) Plate inoculated with a large number of *P. rubens* cells using a platinum hook. (4) Plate inoculated with a small number of *P. rubens* cells using a platinum hook. (b) The inhibition zone diameters on plates (2) and (3) in panel a (*n*=3). w/o: without.

When comparing different cell densities using the platinum hook, a smaller inoculum consistently produced smaller inhibition zones (Fig. 5a). However, when a large number of *Penicillium* cells were inoculated with the platinum hook, the inhibition zone size was comparable to that observed with microspatula inoculation (Fig. 5b).

These results indicate that the optimal method for preparing reference plates involves preculturing the *P. rubens* ATCC 10002 strain on YM agar medium, inoculating the experimental medium using a microspatula and then incubating the plates at 20 °C for ~5 days. This method was used to prepare culture plates shown in the photographs used for the questionnaire (Fig. 1).

### Interview-1

**Fig. 6.**
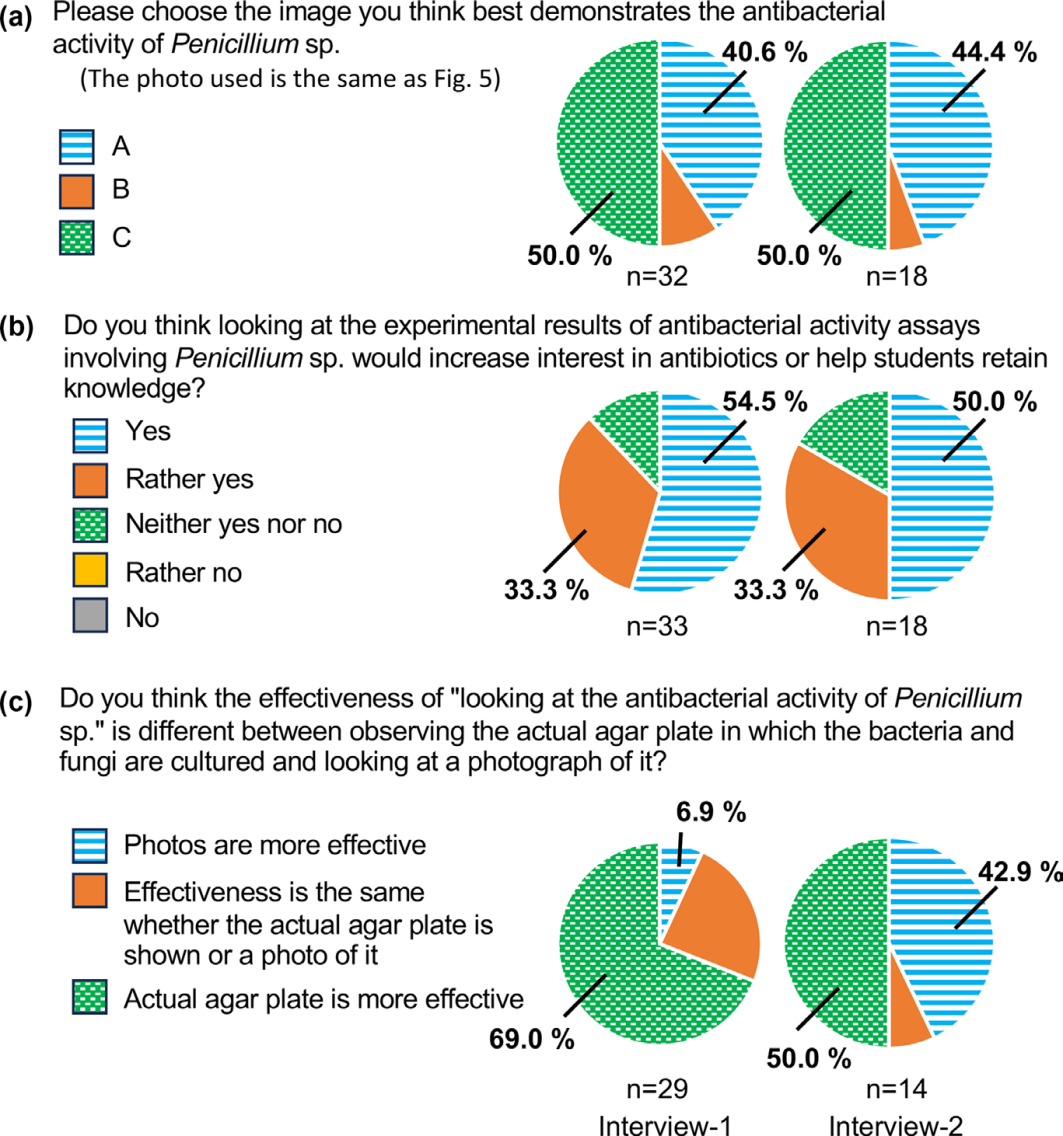
Survey results (partial excerpt). Three survey questions that were common before and after the practical training are compared. For each question shown as A, B and C, the answers before the practical training (%) are shown on the left, and the answers after the practical training (%) are shown on the right in a pie chart. The total number of respondents is shown below the graph.

No students submitted the consent denial or withdrawal form, and 33 students agreed to participate in Interview-1. A survey was conducted among a class of 81 second-year students, with 33 responses. The results of the interview are summarized below (Fig. 6 and Table 1). Before participating in practical training, students were asked whether they had ever seen a photograph or culture plate showing the antibacterial activity of *Penicillium* sp. In total, 51.5% of the students reported having seen antibacterial activity of fungi. Of these, only one student (3.0%) had seen an actual culture plate. Similarly, 45.5% were familiar with the story of penicillin’s discovery. When asked whether visually observing the antibacterial activity of *Penicillium* sp. would have a beneficial effect on their academic performance, 87.9% (29 students) answered that it was beneficial or somewhat beneficial.

These 29 students were then asked whether they perceived a difference in educational effect between photographs and actual plates. Of these, 69% (20 students) responded that the actual plates were more effective (Fig. 6). Additionally, students were shown three photographs of inhibition zones and asked which one they felt best illustrated the effects of antibiotics (Fig. 1). As a result, 50.0% (16 students) selected photo C, while 40.6% (13 students) selected photo A. Because <10% of students found photo B as most effective, samples showing an inhibition zone encircling the entire *Penicillium* colony can be perceived as most effective. Furthermore, 48.5% (16 students) expressed a desire to view actual plates, and another 36.4% (12 students) indicated that they would rather like to see them. These results suggest that providing an opportunity to observe the actual plates during microbiology practical sessions may enhance student engagement.

### Interview-2

No students submitted the consent denial or withdrawal form, and 20 students agreed to participate in Interview-2. A survey was conducted with 69 third-year students after their microbiology practical training, with 20 responses received. The results of the interview are summarized below (Fig. 6 and Table 2). Of the 20 respondents, 18 students had viewed actual plates. These students were shown the same three photographs of inhibition zones as in Interview-1 and asked which photograph they felt demonstrated the strongest antibacterial effect. As in Interview-1, 50.0% (9 students) of the students selected photo C, while 44.4% (8 students) selected photo A. In Interview-2, students who had observed the plates were also asked whether visually observing the antibacterial activity of *Penicillium* sp. had a beneficial effect on their learning. Of the 18 students who had seen the actual plates, 83.3% (15 students) responded that it was beneficial or somewhat beneficial. These 15 students were further asked whether they believed there was a difference in educational effect between photographs and actual plates. Of them, 50.0% (7 students) thought that actual plates were more effective, 42.9% (6 students) believed that photographs were more effective, and 7.1% (1 student) thought that both were equally effective, indicating that the number of students who evaluated the effectiveness of photographs had increased significantly since before the training (Fisher’s exact test: *P*=0.0174).

### Comparison of inhibition circles using multiple *S. aureus* strains

**Fig. 7.**
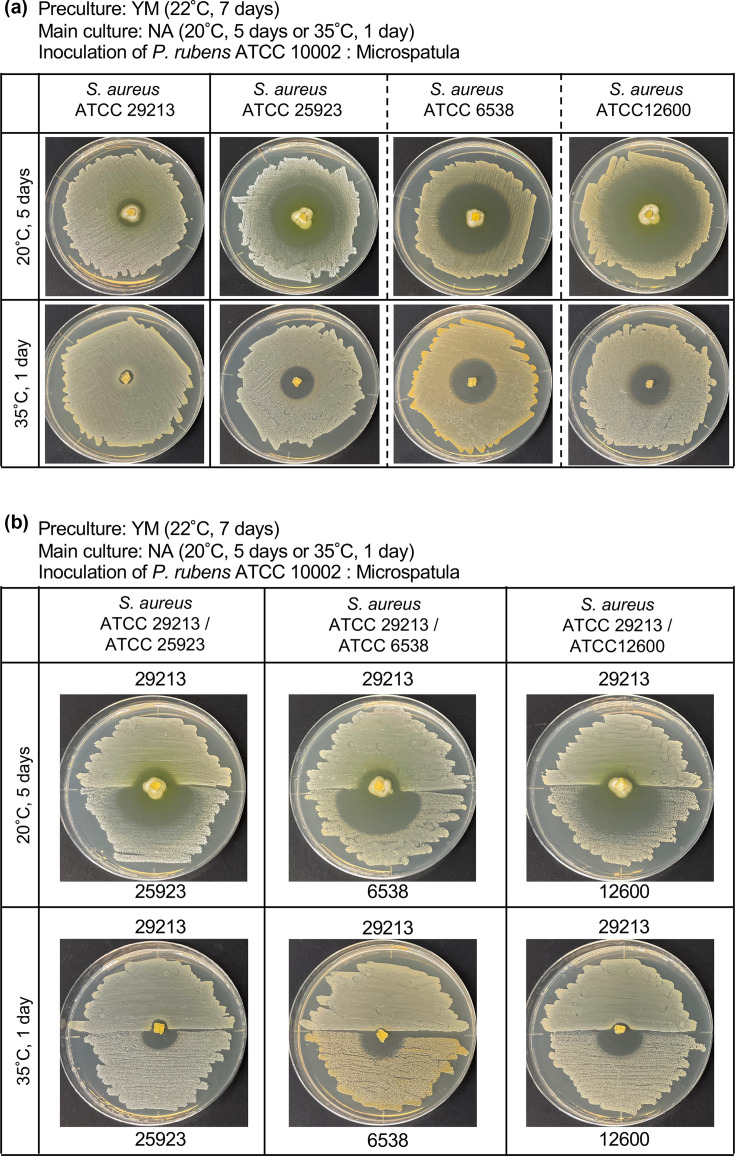
Differences in the size of inhibition zone among *S. aureus* strains and the creation of advanced teaching materials. (a) The size of the inhibition zones against *P. rubens* ATCC 10002 was compared for four *S. aureus* strains. (b) *S. aureus* strains that formed inhibition zones of different sizes were spread onto each half of a plate, and *P. rubens* ATCC 10002 was inoculated into the centre. a and b show a comparison of the inhibition zone after 5 days of incubation at 25 °C and 1 day of incubation at 35 °C.

Fig. 7 shows the results of an experiment using the amount of bacteria used in the EUCAST disc diffusion method. A comparison of the inhibition circles of ATCC 25923, ATCC 6538 and ATCC 12600 against *P. rubens* ATCC 10002 when cultured under different conditions is also shown. After incubation at 35 °C for 1 day, the lactamase-producing ATCC 29213 formed a small inhibition circle measuring 5.83±1.44 mm, whereas ATCC 25923, ATCC 6538 and ATCC 12600 formed circles measuring 17.83±1.53 mm, 22.33±0.76 mm and 20.33±0.29 mm, respectively. These strains showed larger inhibition zones than ATCC 29213 (Fig. 7a). After 5 days of culture at 20 °C, the size of the inhibition zone of ATCC 29213 was 14.33±1.61 mm, whereas that of ATCC 25923, ATCC 6538 and ATCC 12600 was 34.33±1.44 mm, 39.50±3.77 mm and 36.17±0.76 mm, respectively. The double circle of inhibition of ATCC 6538 was mild, whereas ATCC 21600 and ATCC 25923 formed wide double circles of inhibition (Fig. S2C). When cultured at 20 °C, *P. rubens* also grew, creating a visually clear culture plate that shows the inhibition of bacterial growth by fungi.

The results shown in Fig. 7a confirmed the presence of *S. aureus* strains with different susceptibilities to the antibacterial compounds produced by *P. rubens*. Therefore, the *S. aureus* strain that is susceptible to antibacterial compounds and another resistant *S. aureus* strain were streaked on one half, and *P. rubens* ATCC 10002 was inoculated into the centre. Consequently, a plate that allowed us to observe different susceptible strains on one plate was created. In this case, the plates cultured at 20 °C for 5 days were found to be visually effective teaching materials. These photographs of the plate were not used in the student interviews, but they can be used as effective teaching materials that will capture the students’ interest (Fig. 7b). A photograph of the plate without inoculated *P. rubens* is shown in Fig. S2.

## Discussion

On NA, the primary culture medium used in this study, *Penicillium* strains inoculated with a microspatula or platinum hook grew to similar colony diameters in the absence of *S. aureus* (Fig. 2). Despite this, the inhibition zones were often smaller when *Penicillium* cells were inoculated using a platinum hook than when inoculated with a microspatula. However, when a sufficient number of *Penicillium* cells were transferred, a sufficiently large inhibition zone was formed even with a platinum hook (Fig. 5). Although *Penicillium* sp. can grow from a small inoculum, it appears that a certain threshold number of *Penicillium* cells is necessary to produce sufficient antibacterial substances to form an inhibition zone against the bacteria. Microspatula inoculation offers greater consistency as it allows for the even transfer of a larger, uniform amount of *Penicillium* cells. However, platinum hook inoculation often results in variable quantities of cells, which explains the inconsistency in the size of inhibition zones. In addition, when inoculating using a microspatula, some medium is transferred along with the *Penicillium* cells. This could introduce residual antibiotics already produced during preculture, which may influence the formation of the inhibition zone. Therefore, it is possible that the antibiotics present in the transferred medium, combined with the antibiotics newly synthesized by the inoculated *Penicillium* cells, act synergistically to form a larger inhibition zone.

When conducting this experiment using a strain for which penicillin production has not been previously reported, the microspatula method was considered to be more appropriate for initial screening to confirm whether an inhibition zone was formed.

As shown in Fig. 4, multiple colonies were observed within the inhibition zone. *S. aureus* exhibits heteroresistance to some antibiotics [15]. On the contrary, penicillin resistance is induced in *S. aureus* with continued exposure to penicillin. In the absence of penicillin, resistant cells decrease, and they are replaced by sensitive cells [16]. In this experiment, the cells were cultured for 5 days, and resistance may have been induced by continuous exposure to the antibiotic during that period.

This study involved second-year university students who had not yet taken microbiology practical training and third-year university students who had completed it. The small number of participants in Interview-2 is attributed to the limited number of students who advanced from the second to the third year and completed the microbiology practical course. Before the practical course, 33 of 81 students responded, but after the practical course, 20 of 69 students responded. As participation was voluntary, response rates were relatively low.

In contrast, the responses of the questionnaire before the practical training likely reflect perspectives common to individuals, such as junior and senior high school students and the general public, who have little to no hands-on experience in microbiology. For effective infectious disease control, it is essential to provide accurate information about micro-organisms, but sparking initial interest is important. The visual demonstration of antibacterial effects in this study serves as a clear and engaging way to stimulate student interest in micro-organisms. Interestingly, the ranking of photographs that students perceived as most effective remained consistent before and after the practical training – C, A and B (Fig. 1). The number of students who chose photo A or photo C in Fig. 1 indicates that the density of *S. aureus* at which students feel high antibacterial activity varies from student to student, with some students feeling antibacterial activity as greater on plates with lower bacterial density. However, little difference is found between the number of students who chose photo A or photo C, which indicates that using either medium does not affect students’ learning. In the photograph of the plate in which Fleming reported the results of the co-culture of *Staphylococcus* sp. and *Penicillium* sp., *Staphylococcus* sp. was not confluent, and he reported his observations separately for the ‘normal staphylococcal colony’ and the ‘Staphylococci undergoing lysis’ around *Penicillium* sp. [1]. Photo A, which shows a small number of *S. aureus* colonies, could be used to observe the mechanism by which individual colonies respond to antibiotics produced by *Penicillium* sp. On the contrary, when *S. aureus* grows confluently, as shown in photo C, a clear inhibition zone is observed. Therefore, photo A is the closest to reproducing the plate prepared by Fleming, which is a goal of this study, but users can choose to use either photo A or photo C depending on their purpose. In addition, before the practical training, 69.0% of students believed that observing real plates was more effective than viewing photos. However, after the practical training, this number decreased to 50.0%. Instead, after the practical training, the number of students who rated photos as equally or more effective increased from 31.0% to 50.0%. This shift suggests that viewing well-prepared photographs can have a comparable learning effect (Fig. 6). More students rated the photographs potentially because they were nearly identical to the real plates. For example, if the real plate had a larger inhibition circle than the photograph, the percentage of students who rated the real plate may have been higher. In contrast, if the inhibition circle of the photograph was larger, even more students could have rated the photographs. Although this study did not use such photographs in student interviews, multiple *Penicillium* species can be used to compare penicillin-producing and nonproducing strains, and the results can be presented to the students.

In any case, live culture demonstrations are subject to variability owing to differences in microbial growth and experimental accuracy, which might lead to suboptimal results. In contrast, photographs offer consistency, and it is possible to select, store and use photographs of plates with good results, which can provide a more stable learning effect than preparing experimental results using raw medium plates.

Fig. 7 shows an experiment that was conducted to further improve the teaching materials. After 1 day of incubation at 35 °C, all strains formed inhibition zones, but the fungi in the centre did not grow within 1 day, which indicates that the inhibition zones were caused by the antibacterial compounds contained in the transplantation medium (Fig. 7a). According to the list of zone diameter breakpoints (mm) provided by EUCAST, *S. aureus* is resistant to benzylpenicillin if the inhibition zone is smaller than 26 mm [17]. However, at an incubation temperature of 35 °C, which is suitable for bacteria, even penicillin-sensitive strains formed inhibition zones smaller than the breakpoint. Therefore, the fungal growth medium contains fewer antibacterial compounds. This result may be a good teaching material for considering the content of antibacterial compounds. After 5 days of incubation at 20 °C, the fungus in the centre grew, and a large inhibition zone of *S. aureus* against the fungus was formed. Therefore, culturing at 20 °C for 5 days is suitable for reproducing the plate observed by Fleming, although the amount of *S. aureus* cells plated is different. Furthermore, comparing multiple *S. aureus* strains is considered a good teaching material for considering antibiotic resistance.

In this study, the interviews were conducted on pharmacy students, and the pathogenic bacterium *S. aureus* was used. Although not used in this interview, visualizing penicillin-sensitive and -resistant strains on a single plate (Fig. 7b) may provide a more engaging presentation for students. On the contrary, if the goal is simply to observe the inhibition zone, then the biosafety level can be reduced by changing the bacterial species. For instance, *Staphylococcus epidermidis* ATCC 14990 is a BSL1 strain, and the results of using this strain are shown in Fig. S3. *S. epidermidis* did not grow well at 20 °C, with only sparse colonies observed. At 22 °C, however, the strain exhibited adequate growth, and a clear inhibition zone caused by *Penicillium* was also observed. This suggests that when using different bacterial species or strains, culture conditions should be optimized in advance.

## Conclusion

Culture plates were developed to demonstrate the production of antibacterial substances by *Penicillium* sp. and the resulting inhibition of bacterial growth, with the goal of using them as educational material for students in the faculty of pharmaceutical sciences. Among the three fungal strains tested, *P. rubens* ATCC 10002 exhibited the strongest antibacterial activity. Optimal results were achieved when *P. rubens* ATCC 10002 was precultured on YM agar medium, transferred onto the test medium along with the medium and incubated at 20 °C for 5 days, making for good materials as an exhibit. The results of a student interview further supported the educational value of this approach. Observing the antibacterial activity of *Penicillium* was perceived as highly effective in enhancing understanding of microbiological concepts.

The story of the discovery of penicillin is widely known not only in university education but also to the general public, often as the story of a great scientific breakthrough. However, topics such as micro-organisms and antibiotics are rarely emphasized in biology classes at the high school level. The results of this study suggest that visually observing the antibacterial activity of fungi, whether through actual plates or photographs, can be an effective way to stimulate interest. By sharing high-quality photographic materials created at universities, these educational resources can be extended beyond higher education.

## Supplementary material

10.1099/acmi.0.001129.v3 Supplementary Material 1.

10.1099/acmi.0.001129.v3 Uncited Supplementary Material 2.

## Abbreviations

ATCC: American Type Culture Collection
NA: nutrient agar
PDA: potato dextrose agar
PSS: physiological saline solution
YM: yeast mold
