# Supplementary material for: Preparation of culture plates demonstrating anti-Staphylococcus aureus activity of Penicillium sp. and their use as teaching materials
Source: Access Microbiol. 2026 Jun 12;8(6):001129.v3. doi: 10.1099/acmi.0.001129.v3 (PMC13262599; doi:10.1099/acmi.0.001129.v3)
Supplement: Supplementary Material 1. [file acmi-8-01129-s001.pdf]

Supplemental online material

Appendix 1. Questions to students

Our research questions are as follows:

Interview-1

The practical training session on micro-organisms will be conducted in the third year. During the practical training, experimental results regarding the production of penicillin by *Penicillium* sp. will be shown. Please let us know your opinions on the production of antibiotics by fungi.

Please enter an anonymous handle name (your email address or real name will not be collected).

Question 1: The antibiotic "penicillin" was discovered by Fleming. The discovery happened when a fungus grew on an agar plate that had been inoculated with bacteria, and bacterial growth appeared to be inhibited around the fungus. Do you know this story?

1. Yes
2. No

Question 2: Have you ever looked at any evidence demonstrating that *Penicillium* sp. can inhibit the growth of bacteria? Please answer by referring to the example picture.

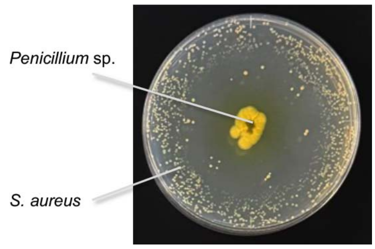

1. Never
2. Only seen in videos or photos
3. Only observed on an actual agar plate
4. Seen on both the actual agar plate and in videos or photos

Question 3: The photos below show the results of an experiment investigating the inhibition of *Staphylococcus aureus* by *Penicillium* sp. *S. aureus* did not grow around the *Penicillium* sp. in the center of the agar plate. Please look at the photos in the top row of A, B, and C and choose the one you think best demonstrates the antibacterial activity of *Penicillium* sp.

1. A
2. B
3. C

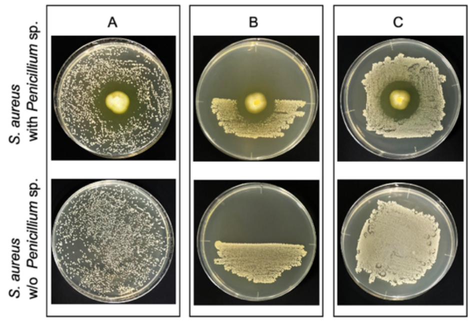

* The bottom photographs in each row are of *Staphylococcus aureus* cultured alone. w/o: without

Question 4: The same photos as in Q3 are shown below. Look at the photos in the top row of A, B, and C, and please choose the photo you think demonstrates the second best antibacterial effect.

1. A
2. B
3. C

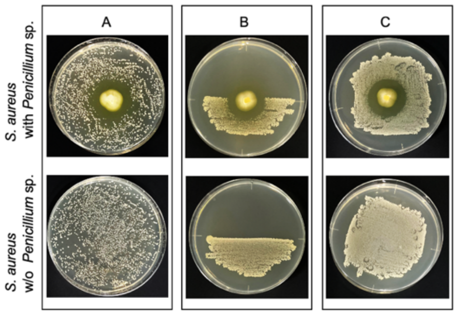

* The bottom photographs in each row are of *Staphylococcus aureus* cultured alone. w/o: without

Question 5: Do you want to look at the actual agar plates used in the experiments shown in Q3 and Q4?
1. Yes
2. Rather yes
3. Neither yes nor no
4. Rather no
5. No

Question 6: Do you think looking at the experimental results of antibacterial activity assays involving *Penicillium* sp. would increase interest in antibiotics or help students retain knowledge?
1. Yes
2. Rather yes
3. Neither yes nor no
4. Rather no
5. No

Question 7: For those who selected "Yes" or " Rather yes" in Q6, do you think the effectiveness of "looking at the antibacterial activity of *Penicillium* sp." is different between observing the actual agar plate in which the bacteria and fungi are cultured and looking at a photograph of it?
1. Photos are more effective
2. Effectiveness is the same whether the actual agar plate is shown or a photo of it
3. Actual agar plate is more effective

Interview-2

Question 1: Do you remember looking at the pictures of agar plates A, B, and C when you were a second-year student?

1. Yes
2. No
3. I didn't answer the questionnaire when I was a second-year student.

Question 2: During your practical training, did you look at the actual agar plates corresponding to A, B, and C?

1. Yes (go to Q3)
2. No (go to Q7)

Question 3: Please choose the one you think best demonstrated the antibacterial activity of *Penicillium* sp.

1. A
2. B
3. C

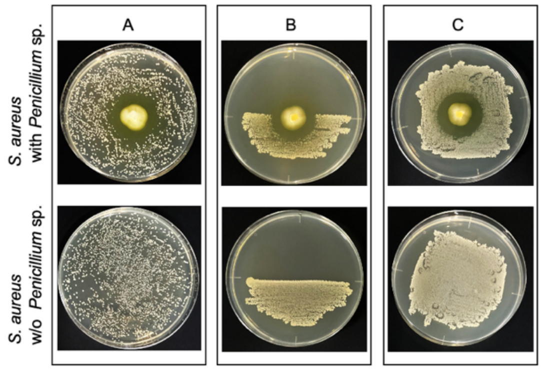

* The bottom photographs in each row are of *Staphylococcus aureus* cultured alone. w/o: without

Question 4: Please choose the photo you think demonstrated the second best antibacterial effect.

1. A
2. B
3. C

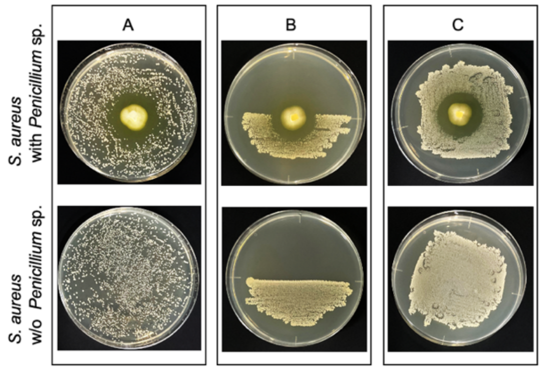

* The bottom photographs in each row are of *Staphylococcus aureus* cultured alone. w/o: without

Question 5: Do you think looking at the experimental results of antibacterial activity assays involving *Penicillium* sp. would increase interest in antibiotics or help students retain knowledge?

1. Yes
2. Rather yes
3. Neither yes nor no
4. Rather no
5. No

Question 6: For those who selected "Yes" or " Rather yes" in Q5, do you think the effectiveness of "looking at the antibacterial activity of *Penicillium* sp." is different between observing the actual agar plate in which the bacteria and fungi are cultured and looking at a photograph of it?

1. Photos are more effective
2. Effectiveness is the same whether the actual agar plate is shown or a photo of it
3. Actual agar plate is more effective

Question 7: For those who did not see the actual agar plates, please tell us why.
