# Supplementary material for: Preparation of culture plates demonstrating anti-Staphylococcus aureus activity of Penicillium sp. and their use as teaching materials
Source: Access Microbiol. 2026 Jun 12;8(6):001129.v3. doi: 10.1099/acmi.0.001129.v3 (PMC13262599; doi:10.1099/acmi.0.001129.v3)
Supplement: Uncited Supplementary Material 2. [file acmi-8-01129-s002.pdf]

SFig.1

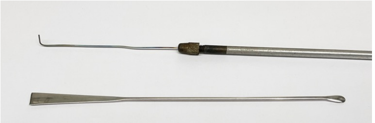

The hook was sterilized by burning sterilization before use. The platinum hook can be made of nichrome wire.

The microspatula should be wrapped in aluminum foil and sterilized by dry heat before use.

Inoculation using the hook

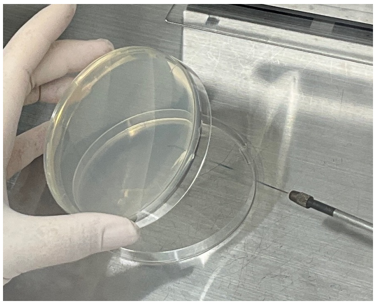

Scraping and inoculating the fungus using the hook.

Using a hook, inoculating from the under of the agar medium reduces contamination by spores.

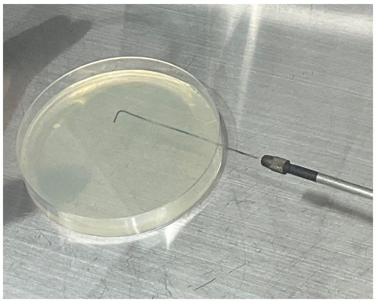

In this study, a substantial amount of fungal cells were inoculated; thus, they were inoculated from the top side of the agar medium.

Inoculation using the microspatula

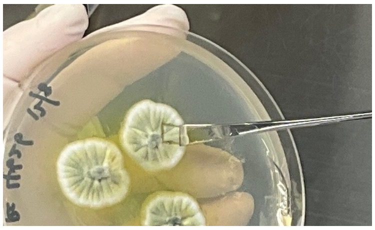

A microspatula was used to press and cut the fungus into squares.

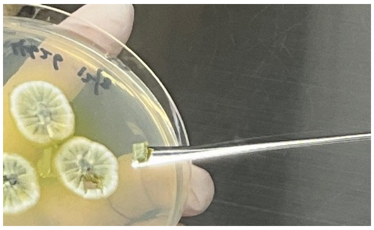

The fungi were placed on the spatula.

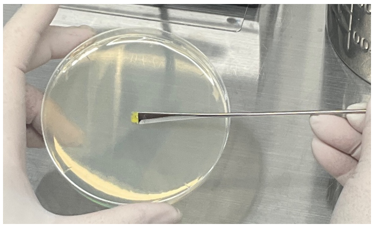

Place the side with the fungi growing on the agar medium.

SFig. 1. Fungal inoculation method
The differences between inoculation methods are shown using an L-shaped platinum colony hook and a microspatula.

SFig.2

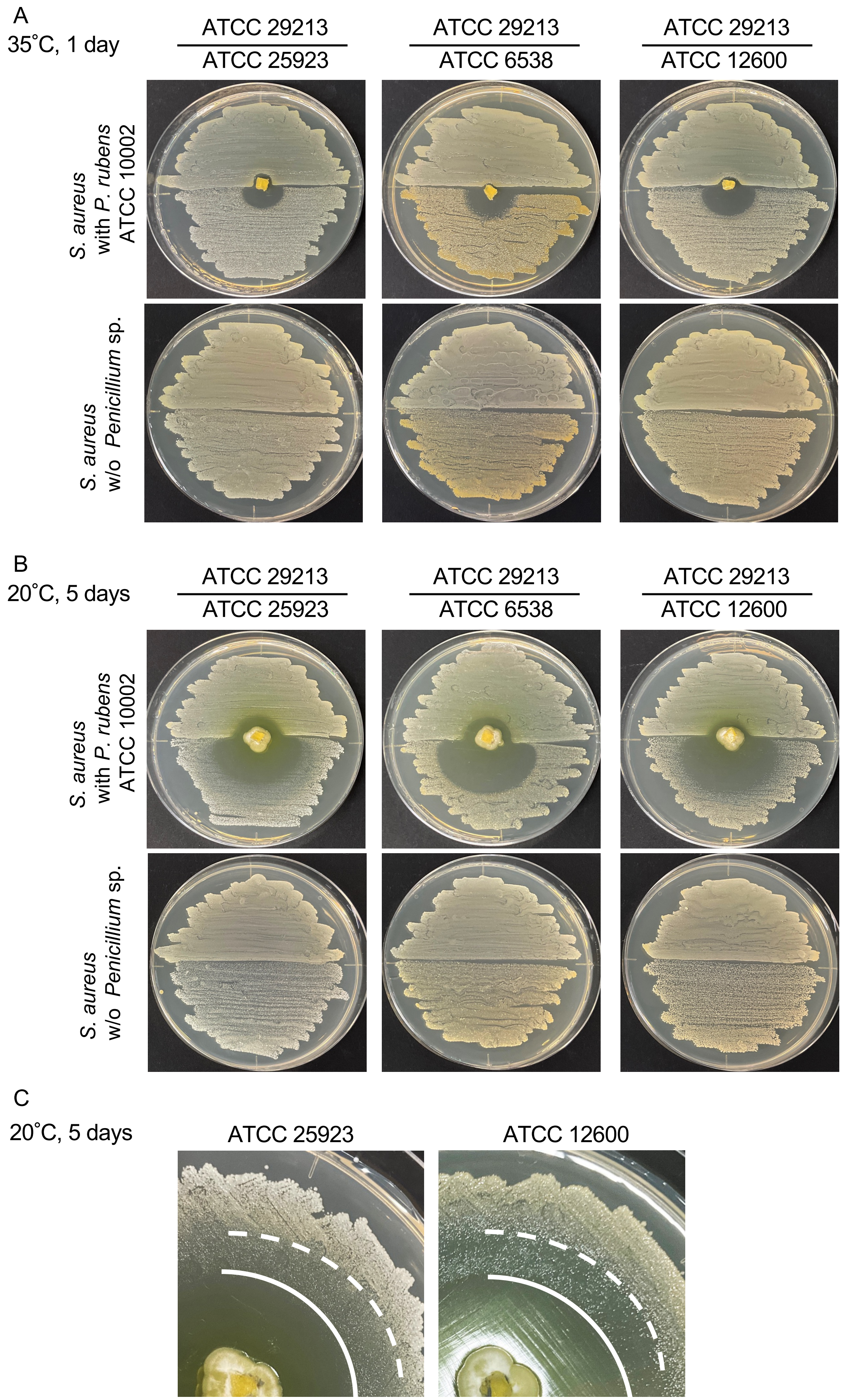

SFig. 2. Supplementary data for Fig. 7.
The inhibition zones were created using different *Staphylococcus aureus* strains on a single plate. ATCC 29213 was spread onto the upper side of the plate, and a penicillin-susceptible strain was spread onto the lower side. (A) Results after 1 day of incubation at 35° C. (B) Results after 5 days of incubation at 20° C. (C) Double inhibition zones of ATCC 21600 and ATCC 25923. The white dotted line indicates the outer zone, and the white line indicates the inner zone.

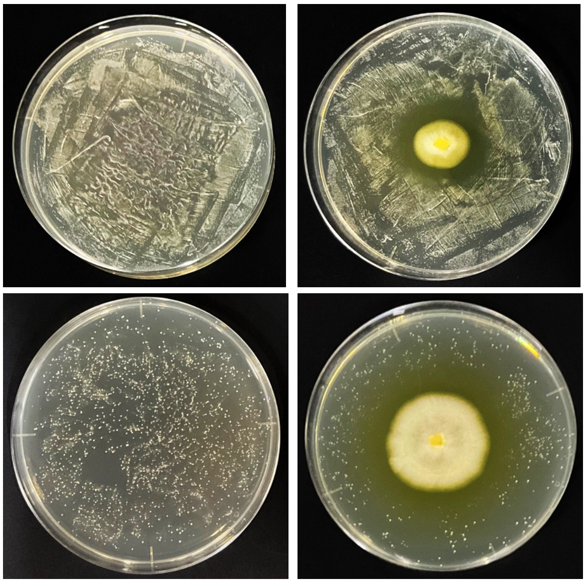

SFig. 3. Inhibition zone against *Staphylococcus epidermidis*

S. epidermidis NBRC 100911 was suspended in physiological saline solution and adjusted to an OD600 of 0.05. This suspension was further diluted fivefold, and 100 µL was spread evenly across the plate using a sterile bacterial spreader. After the medium surface dried, *P. rubens* ATCC 10002 was inoculated at the center of the plate.
